# Supplementary figures and images for: PCBP2 as an intrinsic aging factor regulates the senescence of hBMSCs through the ROS-FGF2 signaling axis
Source: eLife. 2025 Mar 7;13:RP92419. doi: 10.7554/eLife.92419 (PMC11888601; doi:10.7554/eLife.92419)

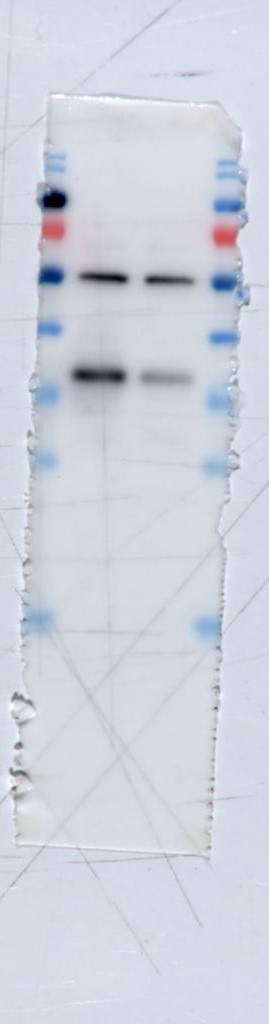

Supplement: Figure 3—source data 1. [file elife-92419-fig3-data1.zip › FIGURE3B-source data 1/Figure 3B-source data 1.jpg]

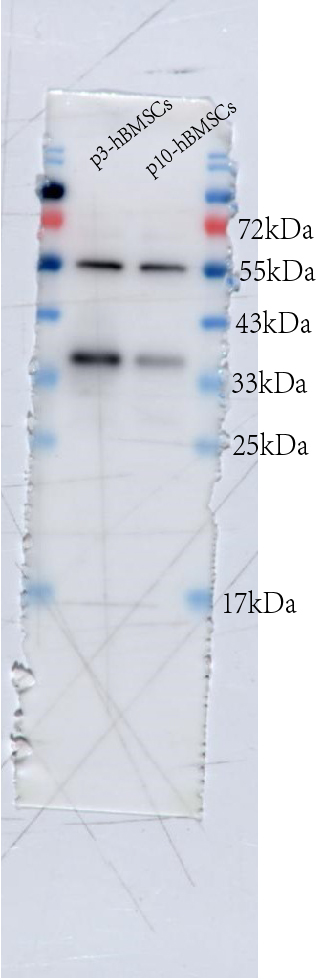

Supplement: Figure 3—source data 2. [file elife-92419-fig3-data2.zip › FIGURE3B-source data 2/Figure 3B-source data 2.jpg]

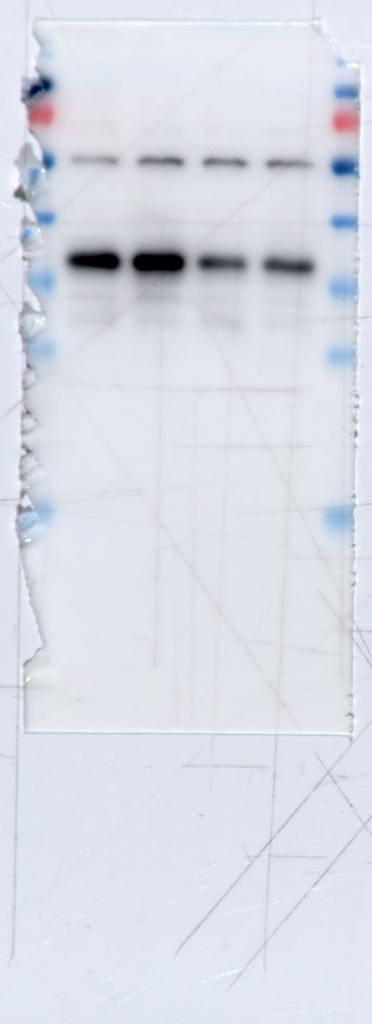

Supplement: Figure 4—source data 1. [file elife-92419-fig4-data1.zip › FIGURE4C-source data 1/Figure 4C-source data 1.jpg]

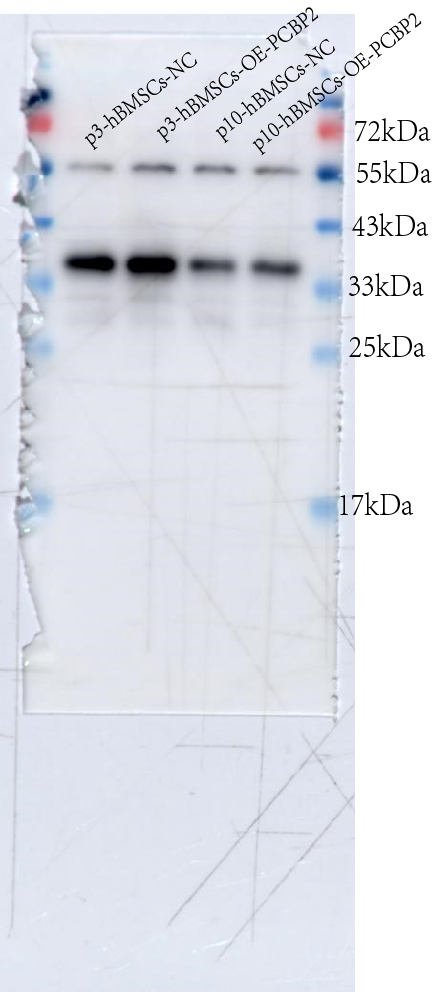

Supplement: Figure 4—source data 2. [file elife-92419-fig4-data2.zip › FIGURE4C-source data 2/Figure 4C-source data 2.jpg]

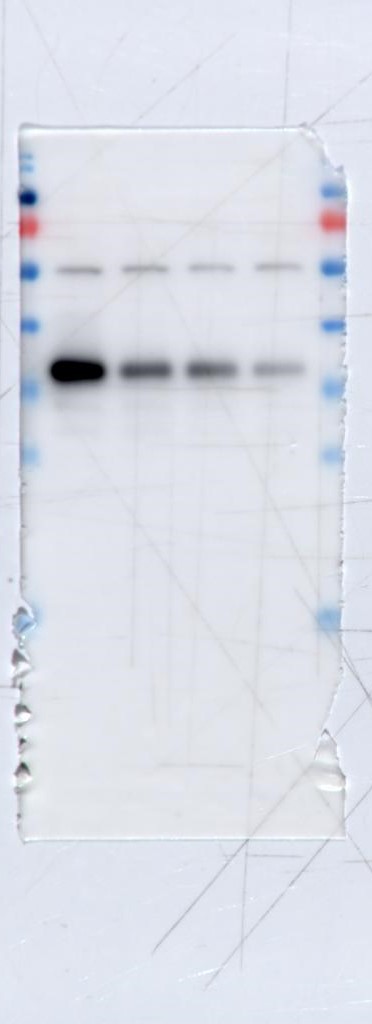

Supplement: Figure 4—source data 3. [file elife-92419-fig4-data3.zip › FIGURE4D-source data 1/Figure 4D-source data 1.jpg]

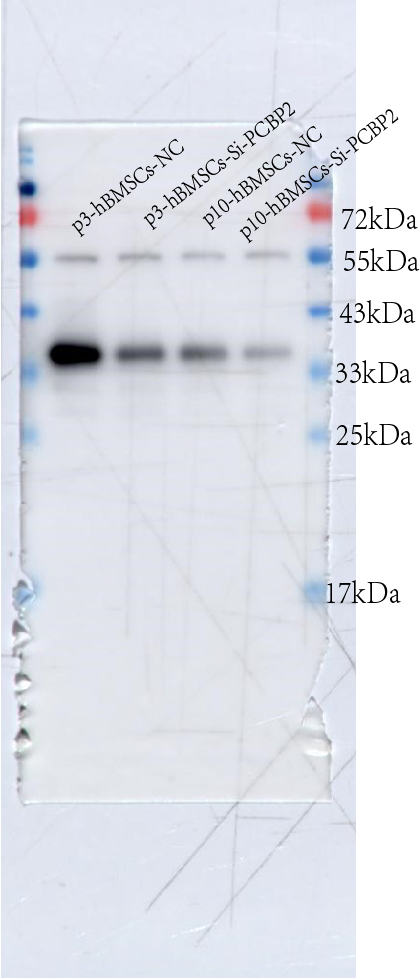

Supplement: Figure 4—source data 4. [file elife-92419-fig4-data4.zip › FIGURE4D-source data 2/Figure 4D-source data 2.jpg]

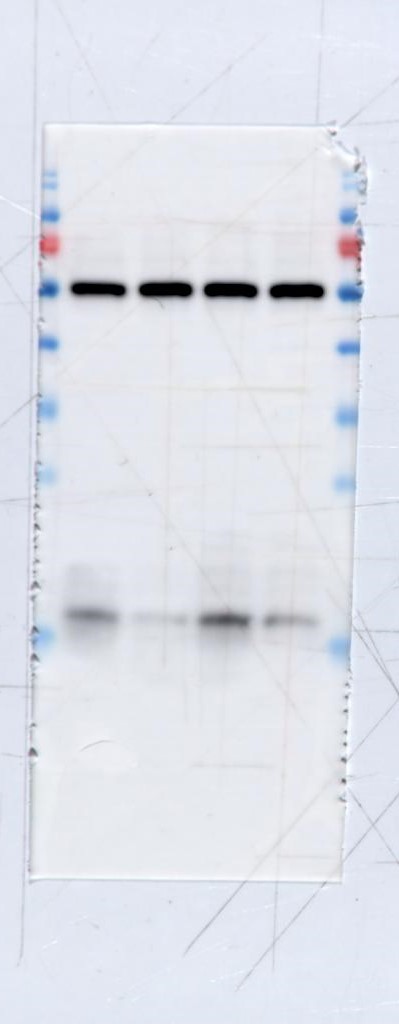

Supplement: Figure 7—source data 1. [file elife-92419-fig7-data1.zip › FIGURE7C-source data 1/Figure 7C-source data 1.jpg]

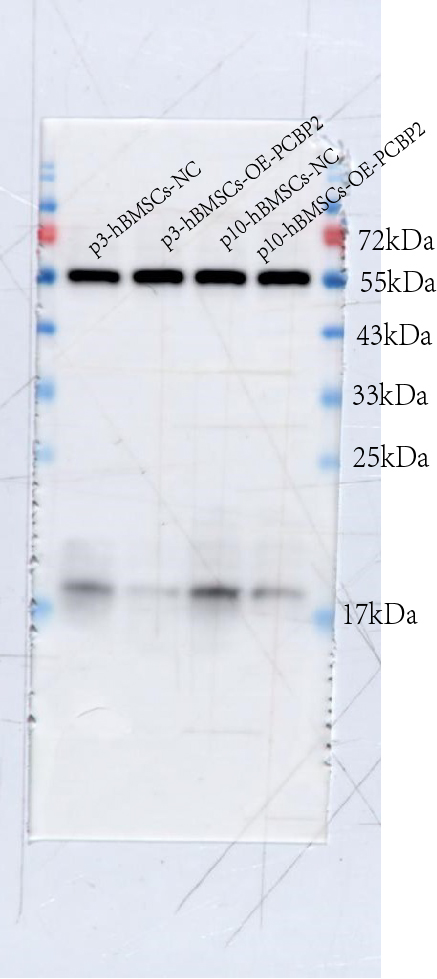

Supplement: Figure 7—source data 2. [file elife-92419-fig7-data2.zip › FIGURE7C-source data 2/Figure 7C-source data 2.jpg]

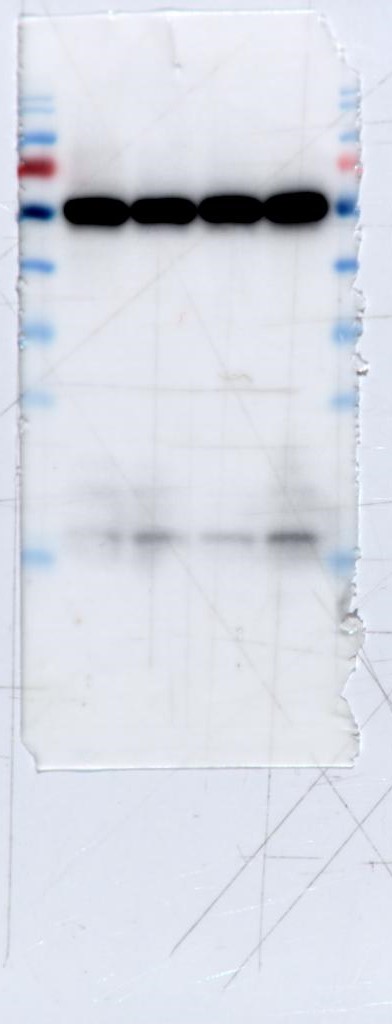

Supplement: Figure 7—source data 3. [file elife-92419-fig7-data3.zip › FIGURE7D-source data 1/Figure 7D-source data 1.jpg]

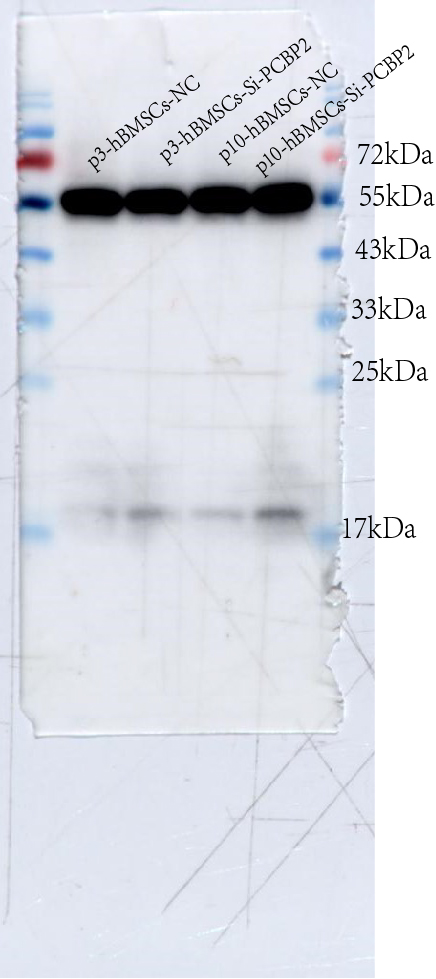

Supplement: Figure 7—source data 4. [file elife-92419-fig7-data4.zip › FIGURE7D-source data 2/Figure 7D-source data 2.jpg]

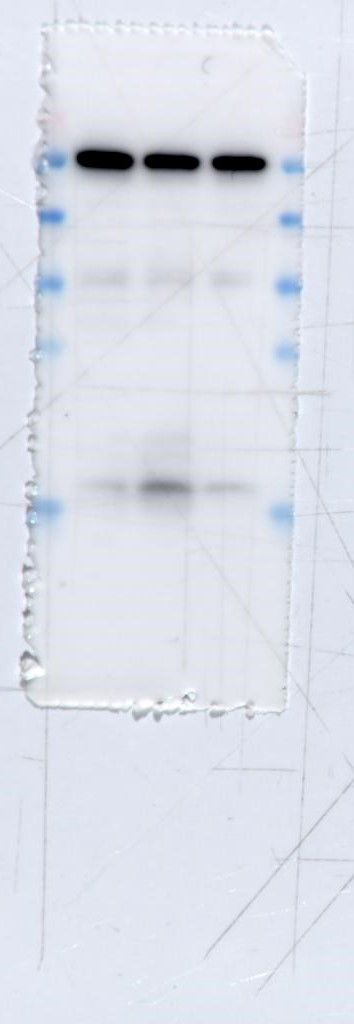

Supplement: Figure 7—source data 5. [file elife-92419-fig7-data5.zip › FIGURE7E-source data 1/Figure 7E-source data 1.jpg]

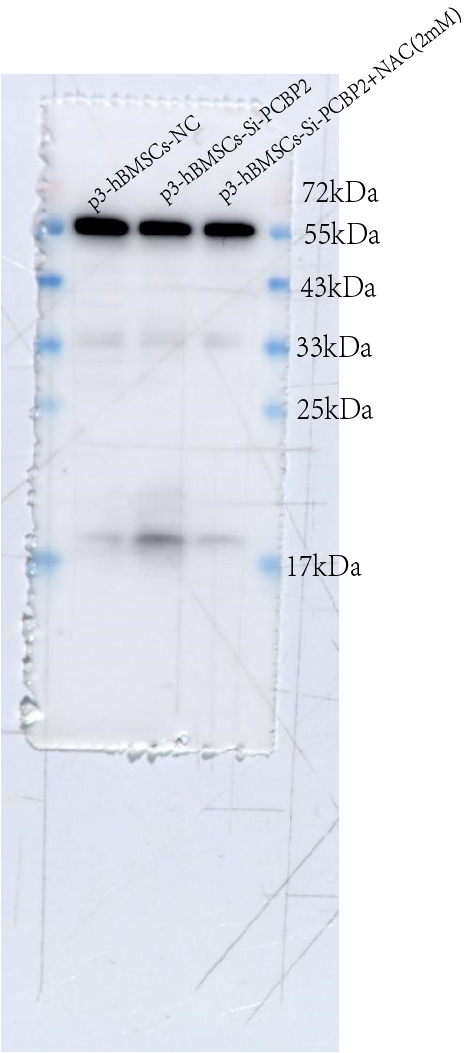

Supplement: Figure 7—source data 6. [file elife-92419-fig7-data6.zip › FIGURE7E-source data 2/Figure 7E-source data 2.jpg]
